# The San Antonio Meeting Texas State Medical Association

**Published:** 1899-04

**Authors:** 


### The San Antonio Meeting Texas State Medical Association.

The annual meeting of the Texas State Medical Association, be it remembered, will be held in the Alamo City the last week in this month, beginning Tuesday, April 25th, and holding four days.

The medical profession are looking forward to the occasion with unusual interest, for there has been a shaking up of dry bones and a revivication going on all along the lines, and it is confidently expected that this will be the "banner meeting" of more recent years; that a "newness of life" will be infused into the organization, and that the profession of the State will be fairly represented by its active membership. It has been sufficiently and repeatedly demonstrated that unless, or rather, until the medical profession becomes unified, nothing can be hoped for or expected in the way of the much needed medical legislation to correct the great evil of quackery in Texas, nor of legislation in the interest of sanitation; and the neglect, failure or refusal of the Twenty-sixth Legislature, now close to the end of its session, to pay any attention to the wishes of the profession as expressed in conversation, by letter and in the drafts of bills which have been submitted, gives additional emphasis to this fact. There is no use in pursuing the same old tactics,—every effort leads to failure. Even now that the "homos," the eclectics and the "physio-medicals,"—whatever they may be,—have been disarmed of any opposition that can be made with any reason or pretext whatever; even now that it has been thought advisable and expedient by the foremost members of the State Association to extend to these parties *recognition* as "schools of medicine," to disarm the objection that any legislation which does not do so is "unconstitutional;" and they *have done so;*—even now, I say, that it has been thought advisable to *come down* from the exalted pinnacle of legitimate medicine and to meet these fellows as equals—before the law,—and they have no longer grounds of opposition—they still have opposed and virtually have defeated the efforts at legislation to "regulate the practice," and to create a board of health. We have humbled ourselves to no purpose, and now chagrin is added to humiliation. (Shake not thy gory locks at me.) Hence, at San Antonio, it is believed that this subject will be discussed, and some plan for the future will be adopted that promises better success. We can only repeat what we have often said: *Organize,* start at the bottom, unite the medical men, and make the question an issue in the next election. If your representative does not "see it," *make* him see it, or, don't vote for him.

The social features of the meeting promises to be a great attraction, and the lady members of the delegates' families and of the families of visiting physicians are invited and expected to be present on the occasion. We publish below the announcement of the Committee of Arrangements and their general invitation. It is extended to every reputable regular physician in Texas.

San Antonio is going to "do herself proud" on the occasion; lay herself out to entertain her guests. The program prepared for the pleasure of guests consists, in addition to other things, of a car ride over the city (for the doctors) late in the afternoon of the second day (Wednesday, 26); a carriage drive for the ladies of the visiting members and delegates; and the famous "Tarpon Club" has extended the privileges of the club for one day to the physicians and their ladies. It is also in contemplation—in fact, in process of completion as we go to press—to have an "excursion"—(our correspondent did not say to what point nor by what means of conveyance) "immediately after the reception" on the third night. A visit to the several "missions" will doubtless be included in the program.

We give below the invitation from the Committee of Arrangements, mentioned above:

San Antonio, Texas, April 3, 1899.

Dear Doctor: We cordially invite and urge you to attend the annual Convention of the Texas State Medical Association, which meets in the quaint and historic city of San Antonio on the 25th of the present month (April), and bring with you your wife and daughters. All indications point to the most important and successful meeting in the history of the Association. The attendance will likely be large beyond precedent, and the papers presented of great practical and scientific interest to the profession. The rates are lower than on any previous occasion, one fare for the round trip, and the local committees have mapped out a program which it is hoped will assure you a pleasant time socially. There seems to be a general awakening of interest in the old Association, and this is as it should be. It is earnestly hoped that the attendance will be not only large, but truly representative of the regular profession of the State. We sincerely hope you will be present, and give the Association the benefit of your active personal co-operation.

San Antonio has hung her latch string on the outside of the door for you. Will you come?

Yours fraternally,
B. E. Hadra,
J. S. Lankford,
B. F. Kingsley,
Amos Graves, Sr.,
Frank Paschal,
James H. Bell,
Committee of Arrangements.

